# Distinguishing ***Bolboceras inaequale*** Westwood, 1848 and two new relatives from India (Coleoptera, Geotrupidae, Bolboceratinae)

**DOI:** 10.3897/zookeys.276.4786

**Published:** 2013-03-08

**Authors:** Jan Krikken

**Affiliations:** 1 Naturalis Biodiversity Center / National Museum of Natural History, PO Box 9517, NL-2300 RA Leiden, The Netherlands

**Keywords:** Coleoptera, Geotrupidae, *Bolboceras inaequale* group, key, new species, India

## Abstract

The taxonomy of the *Bolboceras inaequale* group of species is discussed. The group as here conceived comprises three species in the Indian subcontinent: *Bolboceras inaequale* Westwood, 1848 (reputedly also ranging into sub-Saharan Africa), and two new species: *Bolboceras duplicatum*, and *Bolboceras orissicum*, both from India. All three are keyed, diagnosed, and illustrated; variability and potential taxonomic obstacles are briefly discussed.

## Introduction

Within the mainly South Asian genus *Bolboceras* Kirby, 1819 (nomenclature, cf. ICZN 2006) various operational groups of species can be recognized (Krikken in press). One is the *Bolboceras inaequale* group, comprising a few species with – at least in major individuals – a characteristic set of three pronotal concavities behind and between a transversely arranged double pair of antediscal tubercles (two discoparamedian and two discolateral tubercles, terminology by Krikken (in press)); the discomedian impression is enclosed all around (basin-like), and characteristically shaped in major individuals. The single named species in the group, *Bolboceras inaequale* Westwood, 1848, has been recorded from both the Indian subcontinent and northern sub-Saharan Africa. This paper is intended to show that there is more to the *inaequale* group than this single described species, by discussing the diversity among South Asian group members, while focusing on the identification of two new relatives from India. It is very likely that in the future, based on informative series from more localities, additional taxa will be recognized, while any minor individuals can then be interpreted better than is currently the case; the identity of sub-Saharan material referred to *Bolboceras inaequale* (by Paulian 1941) may also be clarified in the process.

Comparisons of the aedeagi are, as usual in Bolboceratinae, important, but the principle is: externally different aedeagal shapes indicate potentially different species, similar shapes do not necessarily indicate a single particular species. The basic structure and terminology of the aedeagus in *Bolboceras* is illustrated in Krikken (in press).

The *inaequale* operational group is defined below, the second paragraph including the broader generic *Bolboceras* features as applicable to the Asian species.

### Material and methods

For more information on *Bolboceras*, its nomenclature, subdivision into groups, related genera, and technical conventions, cf. Krikken (2013). The list of material examined under *Bolboceras inaequale* follows the original label texts. Specimens and body parts are pictured as is – no remounting, to prevent damage.

### Collections

The material on which this study is based comes from the collections listed hereafter. I most gratefully acknowledge the patient collaboration of the staff concerned.

BMNHThe Natural History Museum, London, UK


BPBMBernice P. Bishop Museum, Honolulu, USA


IRSNBInstitut Royal des Sciences Naturelles de Belgique, Brussels, Belgium


MHNGMusée d’Histoire Naturelle, Geneva, Switzerland


MNHNMuséum National d’Histoire Naturelle, Paris, France


OUMNHUniversity Museum of Natural History, Oxford, UK


RMNHNaturalis Biodiversity Center, Leiden, Netherlands


SMFSenckenberg Museum, Frankfurt, Germany


SMTDStaatliches Museum für Tierkunde, Dresden, Germany


USNMNational Museum of Natural History, Washington DC, USA


ZMHBZoologisches Museum, Humboldt Universität, Berlin, Germany


### The *Bolbocerasinaequale* group

**Group characters.** Upright conical-triangular discoparamedian tubercles on pronotum distinct, more or less approximated, connected by variably wide transverse saddle (watch out for minor morphs); major specimens with broad, well defined discomedian concavity situated (largely) behind these tubercles (shape of its posterior edge varying, arcuate to W-shaped) – minor specimens with similar, more superficial ornamentation; discomedian concavity on either side separated from discolateral concavity by variably elevated saddle (connecting discoparamedian tubercles to posterior disc). Outline of clypeus subtrapeziform (i.e. oblique lateral perimarginal ridges running to anterolateral angles of main surface on either side of transverse anterior ridge, not converging to a single anteromedian tubercle); clypeal surface inside perimarginal ridges without protrusion(s).

Frons with long, distinct, transverse interocular ridge (may reach paraocular ridges on either side). Canthus protuberant, distal tip beyond protuberance arching backward, not reaching temporal area behind eye. Pronotum with set of four distinct tubercles transversely arranged; pronotal base distinctly, completely marginate (ridged). Elytra with 7 shallow punctate striae between suture and humeral umbone, stria 1 ending at sinuate lateral edge of scutellum, stria 2 somewhat effaced near basal scutellar angle, striae 3-7 usually reaching elytral base. Elytral interstrial surface (on cross-section) flat or very slightly convex. Mesocoxae separated by metasternal lobe, intervening space without recurved anterior hook or other isolated protrusions. Antennal lamellae unmodified (lacking grooves, with moderately delimited glabrous area on internal side of lamella 1). Abdominal tip unmodified. Aedeagus with complex median apparatus between (more or less membraneous) parameres; median apparatus usually with pair of sclerotized movable lateral stalks, each just inside parameral sheath (in resting position; note aberrant situation in *Bolboceras duplicatum*). Sexual dimorphism absent or slight. Colour lighter or darker brown, no colour pattern. Body length usually 9–14 mm (5.5–15 mm in other *Bolboceras* groups).

### List of species in the *Bolboceras inaequale* group

*Bolboceras inaequale* Westwood, 1848 – India, Bangladesh; northern sub-Saharan Africa [to be verified, Paulian 1941, Krikken 2011].

*Bolboceras orissicum* **sp.n.** – India: Orissa and Jharkhand.

*Bolboceras duplicatum* **sp.n.** – India: Tamil Nadu.

### Key to species in the *Bolboceras inaequale* group (major individuals)

**Table:** 

1	Transverse interocular ridge not reaching longitudinal paraocular ridge along eyes (Figs 16, 18, 22). Angles on either side of anterior transverse ridge of clypeus strongly elevated, forming U-shaped saddle. Parameres tapering, lateral stalks on median aedeagal apparatus distinct, with tip recurved-hooked (Figs 13–14)	2
–	Transverse interocular ridge reaching paraocular ridge along eyes (Fig. 20). Angles on either side of anterior transverse ridge of clypeus not elevated. Parameres blunt, membraneous, lateral stalks on median aedeagal apparatus indistinct (Fig. 15). Saddle from each discomedian tubercle posteriorly to pronotal disc broad Fig. 21). Tips of pronotal protrusions rounded off	*Bolboceras duplicatum*
2	Discomedian concavity of pronotum with anterior saddle widely separating discoparamedian tubercles, posterior part delimited by simply rounded edge (Fig. 23); lateral saddle short, not lowered (not expanding into adjacent concavity); this lateral concavity posteriorly delimited by sinuate edge. Tips of pronotal protrusions slightly rounded off (Fig. 8)	*Bolboceras orissicum*
–	Discomedian concavity of pronotum situated entirely behind closely approximated discoparamedian tubercles, posteriorly delimited by variably pronounced W-shaped edge (Fig. 17, less obvious in minors, Fig. 19); lateral ridge narrow, lowered, hence more or less “overflowing” to discolateral concavity, which may extend onto anterolateral pronotal corner. Tips of pronotal protrusions (sub)acute (Fig. 2)	*Bolboceras inaequale*

#### Bolboceras inaequale

Westwood, 1848

http://species-id.net/wiki/Bolboceras_inaequale

**Figures 1–8.**
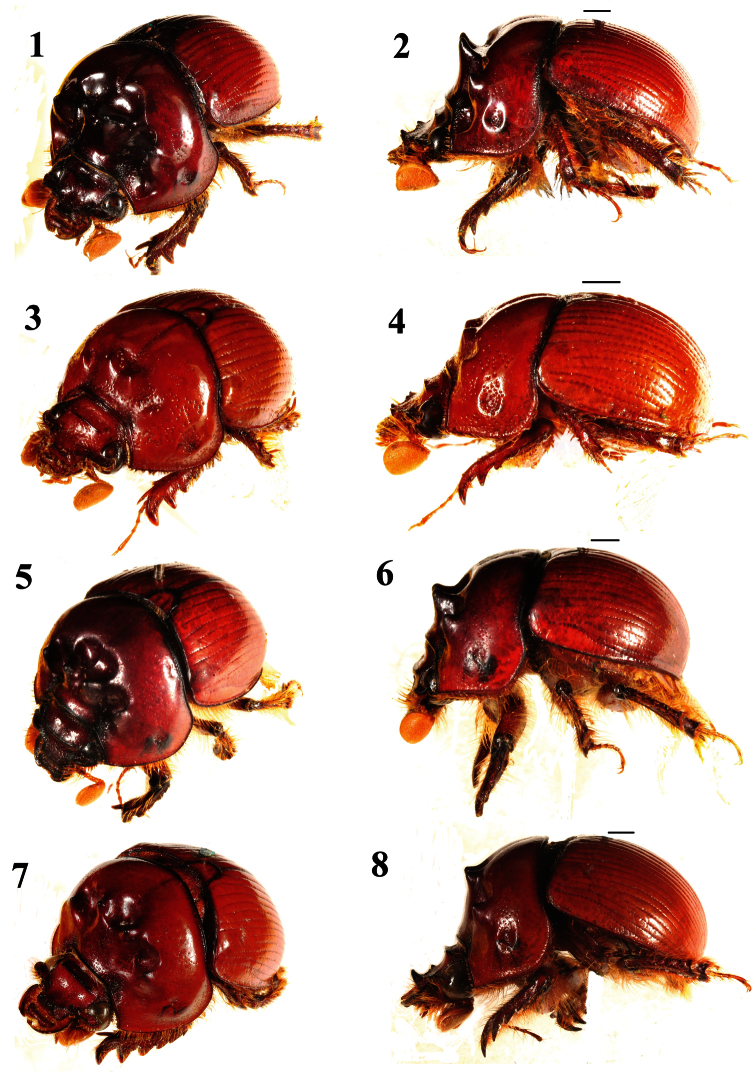
Oblique and lateral habitus views of *Bolboceras*: **1–4** *Bolboceras inaequale* (**1–2** major specimen from Kolkata, length 11 mm **3–4** minor specimen from Dhanjuri, length 9 mm) **5–6** *duplicatum*, holotype **7–8** *orissicum*, holotype. Scale lines 1 mm.

Figures 1–4 9–10, 13 16–19

Bolboceras inaequalis Westwood, 1848: 386 (description); 1952: 24 (description), pl. IV: figs 14, 14a.
Bolboceras inaequale : Paulian 1941: 11, 33 (records from Sudan, Senegal).
Indobolbus inaequalis : Krikken 1984: 34 (recombination in checklist).
Bolboceras inaequale : Krikken 2011: 239, 240 (transfer to original combination).

##### Material examined.

**Syntype,** unsexed, from “Centr Ind \ [J.B.] Hearsey” (handwritten), and two more specimens from Westwood’s collection(OUMNH, see comments below).

**Non-type material (specimens without data excluded).** Bangladesh: Dhaka: Dacca [Dhaka], ex Pascoe coll., 1 spm., in BMNH. Dinajpur: Dhanjuri, v/1963, Mapelli, 2 spm., in MHNG. vi/1963, Mapelli, 2 spm., in MHNG. Rajshahi: Andharkota, vii/1963, Mapelli, 1 spm., in MHNG.

India: Bihar: Buxar, ex Felsche coll., 1 spm., in SMTD. Chapra, Mackenzie, 6 spm., in BMNH. Pusa, 03/viii/1926, Pillai, at light, 1 spm., in BMNH. Pusa, 1913, Fletcher TB, 1 spm., in BPBM. 19/vi/1911, TMH, 1 spm., in BMNH. 30/vii/1915, Bahadur U, at light, 1 spm., in BMNH. 28/vii/1928, Sarkar SC, 1 spm., in BMNH. 20/vii/1908, TNS, at light, 1 spm., in BMNH. 24/vii/1919, Austin GD, verandah 1-10 PM, 1 spm., in BMNH. 08/vii/1915, at light, 1 spm., in BMNH. 12/vii/1915, at light, 1 spm., in BMNH. 05/vii/1924, Mukerjee, 1 spm., in BMNH. 31/vii/1918, Ghosh, underground, 1 spm., in BMNH. 15/viii/1908, RDD, at light, 1 spm., in BMNH. 20/viii/1919, Austin GD, 1 spm., in BMNH. 08/ix/1912, NML, 1 spm., in BMNH. 27/ix/1915, Bahadur U, at light, 1 spm., in BMNH. 04/ix/1915, Bahadur U, at light, 1 spm., in BMNH. 04/x/1915, Bahadur U, at light, 1 spm., in BMNH. 18/xi/1920, Sarkar SC, 1 spm., in BMNH. Tamil Nadu: Madras [Chennai; to be confirmed], ex Felsche coll., 2 spm., in SMTD. Uttar Pradesh: Allahabad, ex Bowring coll., 1 spm., in BMNH. Sitapur, HGC [Champion HG], 4 spm., in BMNH. West Bengal: Calcutta [Kolkata], 1 spm., in BMNH. Kalkutta [Kolkata], ex Felsche coll., 5 spm., in SMTD.

Bengal: unspecified, Parish HM, 1 spm., in BMNH. unspecified, ex Gillet coll., 1 spm., in IRSNB.

India: “Inde”, unspecified, ex Boucomont coll., 1 spm., in MNHN. unspecified, 1 spm., in USNM.

Total 47 males and females, 33 collection records, and Westwood’s material.

##### Diagnosis.

Ridge (or saddle) separating discomedian and discolateral concavities on pronotum narrow, lowered in relation to surrounding discal surface; discoparamedian tubercles approximated (connected by saddle), their tip subacute; discolateral concavity in major males extending down, onto anterolateral surface. Posterior edge of discomedian concavity usually distinctly W-shaped in major individuals, in minors the pronotal ornamentation may be only just distinct (in oblique view); bottom of concavities more or less sericeous. Interocular ridge low, not reaching paraocular ridge on either side, situated between posterior part of eyes. Anterolateral corners of clypeus usually protuberant (in majors intervening ridge concave in axial view). Scutellum usually closely punctate. Intermesocoxal lobe of metasternum anteriorly with arcuate ridge. Aedeagus (Fig. 13) with acuminate parameres, median apparatus with pair of strongly sclerotized lateral stalks, their tip broad, shortly recurved-hooked. Colour uniformly brown. Body length usually 11-13, some series down to ca 9 mm or less (see next section).

##### Variation.

There are small individuals (minors) from Northeast India and Bangladesh (a male from Dhanjuri in Bangladesh pictured here, ca 9 mm long, Figs 3–4, 10, 18–19), characterized by a minimal set of pronotal protrusions and impressions, but still with distinct traces of the basic pattern – and also with a smaller, but externally quite similar aedeagus. Some intermediates between these minors and the real majors (including the type) were seen. At this moment I find no morphological support for ranking the minors as a separate taxon – informative material from different locations required. Paulian (1941) mentions a length range for *Bolboceras inaequale* of 7–12 mm.

##### Sexual dimorphism.

Apparently no notable sexual dimorphism.

##### Comments.

Note the recent retransfer of this species from *Indobolbus* Nikolajev, 1979, to *Bolboceras* Kirby, 1819, and the ensuing suffix change of the species-group name, following the ICZN (2006) ruling. The specimen of *Bolboceras inaequale* labelled as the type (OUMNH) cannot be considered the holotype, as there are two more historical specimens having the usual large (pale blue) rhomboid label with “W” (meaning Westwood’s original collection), one of them with this label only. The specimen here qualified as syntype has a curatorial label of the 1960s, indicating its registration as Coleoptera type 516, plus additional post-Westwood labels. The three specimens are considered conspecific, and consequently, at this moment, an explicit lectotype designation appears unnecessary.

##### Distribution.

India (southern occurrences to be confirmed), Bangladesh; northern sub-Saharan Africa (needs confirmation).

#### Bolboceras duplicatum sp. n.

urn:lsid:zoobank.org:act:DDE7EE13-C2C5-4187-9CF5-1B290729ACDE

http://species-id.net/wiki/Bolboceras_duplicatum

Figures 5–6 11, 15 20–21

##### Material examined.

**Holotype** male (RMNH) from South India: Madras [Chennai], x.1975, T.R.S. Nathan.

##### Diagnosis.

Saddle separating discomedian from discolateral concavities on pronotum short, thick, slightly lowered in relation to surrounding surface; discoparamedian tubercles approximated, but less than in the preceding species; discolateral concavity in no way extending onto anterolateral pronotal declivity. Interocular transverse ridge low, reaching paraocular ridge on either side, about halfway eye. Posterior edge of discomedian concavity only vaguely W-shaped. Pronotal tubercles with rounded tip. Anterolateral corners of clypeus appearing not strongly protuberant. Scutellum sparsely, finely punctate. Aedeagus unusual for *Bolboceras*: with blunt, membraneous parameres, the median apparatus lacking projecting sclerotized lateral stalks. Colour uniformly medium-brown. Body relatively large (length assumed roughly 10–11 mm).

##### Description

(holotype, male). Body length ca 11.5 mm. Colour uniformly medium-brown, shiny (surface locally worn, dull).

Labrum with vaguely emarginate anteromedian border, transverse ridge on coarsely punctate upper surface distinct. Clypeal surface with supra-anterolateral angles distinct, not raised (worn), intervening transverse anterior ridge very slightly convex in full-face view, almost straight; lateral perimarginal ridges distinct, moderately evenly curved, genal angle distinct. Clypeus densely, unevenly, distinctly punctate, frons abundantly, more finely punctate; secondary punctation on frons sparse. Anterior edge of eye canthus thickened-raised to slight arcuate-raised edge of short distal lobe; surface coarsely rugulate-punctate; paraocular ridge distinct, starting at genal angle, virtually straight, extending posteriorly along eye. Transverse interocular elevation situated halfway to eyes (in dorsal view), long, low, distinctly reaching paraocular ridges, its lateral slope slight, crest narrow, unmodified, lateral angle obsolete on either end (axial view).

Pronotum anteromedially steeply declivous, shortly depressed at base (lateral view), outline of marginate (raised) anterior border convex (dorsal view); discoparamedian and discolateral protrusions on pronotum distinctly protuberant, their tip rounded, particularly in discoparamedians (axial view); discomedian concavity very distinct, broad, largely situated behind closely set discoparamedian tubercles (not simply continuing over anterior declivity), posterior edge slightly bisinuate (dorsal view); discolateral concavity deep, posterior edge rounded, anteriorly delimited by crowdedly, coarsely punctate patch behind eyes; saddle from discoparamedian tubercle to posterior disc broad, slightly lowered; bottom of discal concavities more or less matt; basomedian surface with sparsely punctate midline impression; anterolateral angle of marginal pronotal ridge ca 100˚ (full-face view). Pronotal surface with double punctation, primary punctation (size variable) laterally generally abundant; secondary punctation sparse, minute. Pronotal base broadly marginate, lined with row(s) of fine punctures. Scutellum with scattered, sparse double punctation.

Elytra with discal striae shallowly impressed, finely punctate; punctures separated by 3-5 puncture diameters, slightly crenulating interstriae (striae 2 and 5 slightly effaced in front). Elytral interstriae (on cross-section) very slightly convex, vaguely, sparsely, micropunctate.

Intercoxal anterior lobe of metasternum simply truncate in front.

Protibia with 6 external denticles (tips worn off); apex unmodified, with robust, complanate, slightly tapering spur. Outher side of meso- and metatibiae with bilobate apical and one complete anteapical fossorial elevation (their crest fringed with fine spines).

Aedeagus, Fig. 15; lateral stalks apparently absent, parameral sheaths membraneous, with blunt, folded tip.

Measurements of body parts. Median length of head (full-face, excluding labrum and mandibles) 2.5 mm, width 3.6 mm. Median length of pronotum (dorsal) 4.2 mm, maximum width 7.1 mm. Median length of scutellum 1.2 mm, maximum width 1.4 mm. Sutural length of elytra (dorsal) 4.1 mm, maximum width combined 7.2 mm. Width of genital capsule 1.20 mm.

##### Variation.

Only one male seen – beware of possibly deceptive polymorphism.

##### Distribution.

Southeast India.

##### Etymology.

Name refers to its similarity to the preceding species.

**Figures 9–15.**
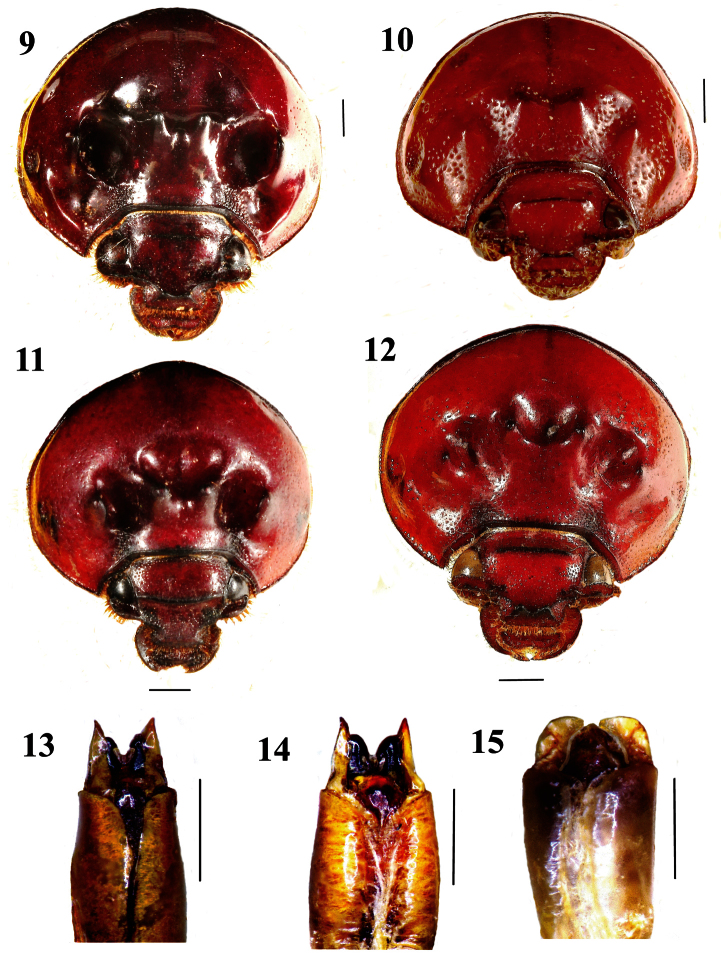
Forebody and aedeagus of *Bolboceras*: **9–12** head and pronotum, frontodorsal view **9–10** *Bolboceras inaequale* (**9** major specimen from Kolkata **10** minor specimen from Dhanjuri) **11** *duplicatum*,holotype **12** *orissicum*,holotype **13–15** aedeagus, upper side view, of **13** *Bolboceras inaequale*,major specimen from Kolkata **14** *orissicum*,holotype **15** *duplicatum*,holotype. Scale lines 1 mm.

#### Bolboceras orissicum sp. n.

urn:lsid:zoobank.org:act:64EA91B5-7AB9-4EE0-BAF7-33C24FCB6BE3

http://species-id.net/wiki/Bolboceras_orissicum

Figures 7–8 12, 14 22–23

##### Material examined.

**Holotype** male (MNHN, ex Boucomont coll.) from India: [Orissa:] Ganjam: Surada [also spelled Sorada], H. Donckier. Male and female paratypes (SMF) from [India: Jharkhand:] “Burju \ Bengal”.

##### Diagnosis.

Saddle separating discomedian and discolateral concavities on pronotum very short, broad, not lowered in relation to surrounding discal surface, discoparamedian tubercles distinctly separated by anterior part of discomedian concavity between them; discolateral concavity quasi-extending onto shallowly concave anterolateral corner; posterior edge of discolateral concavity well defined, “swollen” near saddle with discomedian concavity (outline in dorsal view sinuate). Posterior edge of discomedian concavity virtually rounded. Interocular transverse ridge situated between posterior part of eyes, low, not reaching paraocular ridge on either side. Anterolateral corners of clypeus strongly protuberant (intervening ridge concave in axial view). Scutellum usually abundantly punctate. Aedeagus with narrow, acuminate parameres, median apparatus with pair of strongly sclerotized lateral stalks, their tip very broadly recurved-hooked. Colour uniformly light-brown. Body length roughly 12.5–13.5 mm.

##### Description

(holotype, male). Body length ca 13 mm. Colour uniformly light-brown, shiny, certain parts sericeous.

Labrum with emarginate anteromedian border, transverse ridge on rugulate upper surface fine, distinct. Clypeal surface with supra-anterolateral angles distinctly raised, dentate, intervening ridge concave in axial view, straight in full-face view; lateral perimarginal ridges distinct, strongly evenly curved, genal angle distinct. Clypeus densely punctate, remainder of head surface abundantly, evenly, distinctly punctate, primary punctures interspersed with fine secondary punctation being denser behind interocular ridge. Anterior edge of eye canthus raised to slight anterolateral angle, thence arcuate along edge of distal lobe; surface coarsely rugulate; paraocular ridge distinct, fine issuing from genal angle, virtually straight, extending posteriorly along eye. Transverse interocular elevation between posterior part of eyes, long, low, not reaching paraocular ridges, lateral slope slight, crest fine, unmodified, lateral angle obtuse on either end (axial view). Much of head surface subsericeous.

Pronotum anteromedially steeply declivous, outline of marginate (raised) anterior border slightly convex (dorsal view); discoparamedian and discolateral protrusions on pronotum distinctly protuberant, subrectangular (lateral view), their tip rounded; discomedian concavity very distinct, evenly concave, broad, anterior part situated between discoparamedian tubercles (not continuing over anterior declivity), posterior edge broadly rounded (dorsal view); discolateral concavity deep, posterior edge sinuate; anteriorly, at bottom, delimited by crowdedly, coarsely punctate patch behind eyes; saddle from discoparamedian tubercle to posterior disc very short, not lowered; bottom of discal concavities and anterior declivity subsericeous; basomedian surface glossy, with abundantly, finely punctate midline impression; anterolateral angle of marginal pronotal ridge ca 100˚ (full-face view). Pronotal surface with double punctation, primary punctation (size variable) laterally generally abundant, denser on anterior declivity; secondary punctation sparse, minute. Pronotal base broadly marginate, lined with numerous punctures. Scutellum with abundant, double punctation.

Intercoxal anterior lobe of metasternum simply truncate in front.

Elytra with discal striae shallowly impressed, finely punctate; punctures separated by 2-3 puncture diameters, slightly crenulating interstriae. Elytral interstriae (on cross-section) very slightly convex, vaguely, sparsely, micropunctate.

Protibia with 6 external denticles; apex unmodified, with robust, complanate, slightly tapering spur. Outer side of meso- and metatibiae with bilobate apical and one complete anteapical fossorial elevation (their crest fringed with fine spines).

Aedeagus, Fig. 14; sclerotized lateral stalks broad with recurved, hooked tip, parameral sheaths narrow, acuminate.

Measurements of body parts. Median length of head (full-face, excluding labrum and mandibles) 2.6 mm, width (including eyes) 4.2 mm. Median length of pronotum (dorsal) 4.6 mm, maximum width 8.2 mm. Median length of scutellum 1.1 mm, maximum width 1.4 mm. Sutural length of elytra (dorsal) 3.9 mm, maximum width combined 8.3 mm. Width genital capsule 1.35 mm.

##### Variation.

Variation slight, but beware of possibly deceptive polymorphism (may be obvious in larger series).

##### Sexual dimorphism.

No obvious sexual dimorphism.

##### Distribution.

Northeast India, apparently South of the Ganges.

##### Comment.

The place called Burju, origin of the paratypes, is mentioned in the list of “post-office pincodes” in the Ranchi District of Jharkhand (India), and at the time of the collection of the SMF specimens there was a German mission school in the area, with staff sending specimens to German entomologists (around 1890–1910).

##### Etymology.

Named after the type region.

**Figures 16–23.**
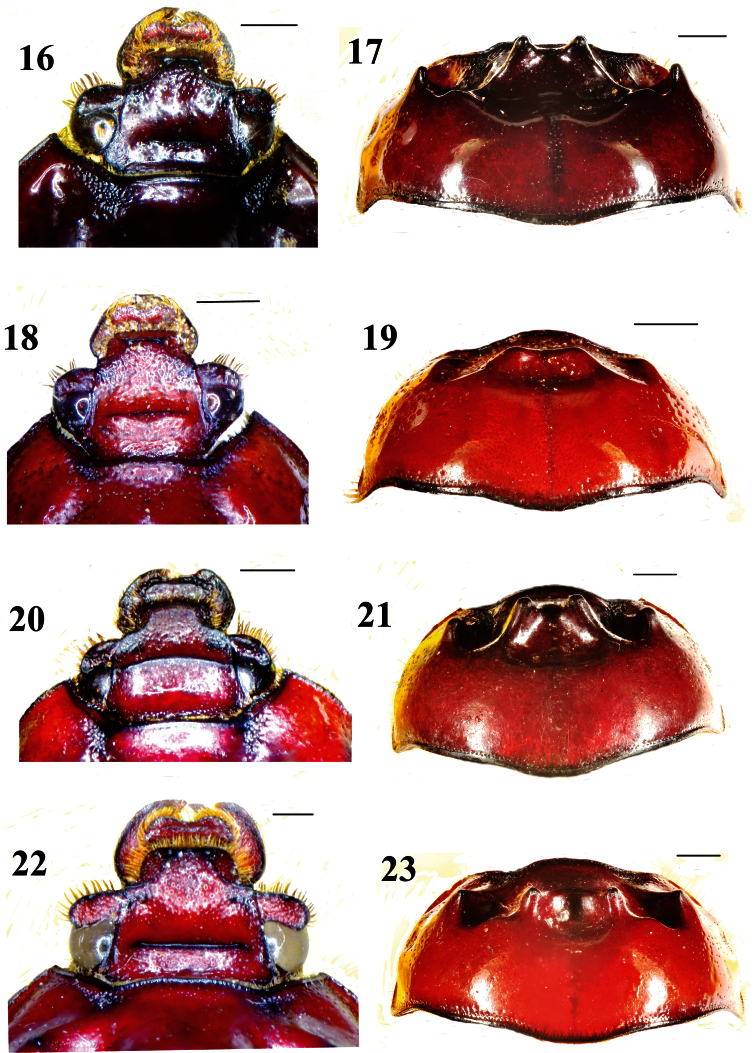
Head, full-face view, and pronotum, dorsal view, of *Bolboceras*: **16–19** *Bolboceras inaequale* (**16–17** major specimen from Kolkata **18–19** minor specimen from Dhanjuri) **20–21** *duplicatum*,holotype **22–23** *orissicum*,holotype. Scale lines 1 mm.

## Supplementary Material

XML Treatment for Bolboceras inaequale

XML Treatment for Bolboceras duplicatum

XML Treatment for Bolboceras orissicum
